# Isolation and characterization of a *Bdellovibrio bacteriovorus* from fish pond water

**DOI:** 10.3389/fmicb.2024.1479942

**Published:** 2024-12-11

**Authors:** Qian-Ming Hong, Kai Yuan, Meng-En Zhang, Xin-Rui Yang, Qi Chen, Shi-Wei Xu, Wan-Yi Chen, Shi-Xin Qian, Yu-Tao Miao, Zhi-Ming Zhu, Yi-Hong Chen

**Affiliations:** ^1^Institute of Modern Aquaculture Science and Engineering (IMASE), Key Laboratory for Healthy and Safe Aquaculture, College of Life Science, South China Normal University, Guangzhou, China; ^2^Southern Marine Science and Engineering Guangdong Laboratory (Zhuhai), Zhuhai, China; ^3^School of Life Science, Huizhou University, Huizhou, Guangdong, China; ^4^Guangdong Provincial Observation and Research Station for Marine Ranching in Lingdingyang Bay, China-ASEAN Belt and Road Joint Laboratory on Mariculture Technology, Zhuhai, China

**Keywords:** *Bdellovibrio bacteriovorus*, aquaculture, disease, pathogenic bacteria, aquaculture effluent treatment, lysed properties

## Abstract

**Introduction:**

The intricate habitats of aquatic organisms, coupled with the prevalence of pathogens, contribute to a high incidence of various diseases, particularly bacterial infections. Consequently, the formulation of sustainable and effective disease management strategies is crucial for the thriving aquaculture sector.

**Methods and results:**

In this investigation, a strain of *Bdellovibrio bacteriovorus*, designated *B. bacteriovorus FWA*, was isolated from a freshwater fish pond. Identification was achieved through microscopic examination of morphological characteristics, biochemical property assessment, and phylogenetic analysis. The lysogenic capability of *B. bacteriovorus FWA* was evaluated, revealing its effectiveness in lysing *Escherichia coli*, *Aeromonas hydrophila*, *Vibrio alginolyticus*, *Vibrio parahaemolyticus*, and *Edwardsiella tarda*. Physiological analysis indicated that the optimal ratio of *B. bacteriovorus FWA* to host bacteria was 1:10,000, with strict aerobic requirements. The optimal pH range for growth and reproduction was 7.0–8.0, the ideal temperature was found to be 30–35°C, with a preferred Na^+^ concentration of 0% and a Ca^2+^ concentration of 15–25 mM. Additionally, *B. bacteriovorus FWA* demonstrated enhanced lytic activity against bacteria in aquaculture effluent while effectively managing ammonia-nitrogen levels.

**Discussion:**

In summary, *B. bacteriovorus FWA* holds significant promise for development as a probiotic agent in aquaculture.

## 1 Introduction

In recent decades, the aquaculture industry has made significant contributions to providing high-quality animal protein to humans. According to data from the Food and Agriculture Organization of the United Nations (FAO), the global volume of aquatic products from 1959 to 2021 is increasing year by year. Based on preliminary statistics, the global volume of aquatic products in 2021 is approximately 218 million tons (FAO, 2024). In the future, aquaculture will continue to be the fastest growing field of animal food production, which is an important guarantee for national food security.

Although the aquaculture industry is important to human society, the problem of disease has plagued its healthy development. Most common aquatic animal diseases are caused by a variety of pathogenic aquatic animal organisms. These pathogenic organisms are called pathogens (Hutson et al., 2023; Nguyen, 2024). Pathogens of aquatic animals mainly include fungi, viruses, bacteria, algae, protozoa, worms, leeches, and crustaceans. Bacterial diseases are particularly prevalent in aquaculture, predominantly caused by Gram-negative bacteria such as *Edwardsiella*, *Vibrio*, *Pseudomonas*, *Aeromonas*, and *Acinetobacter* (Lieke et al., 2020; Lafferty et al., 2015). These bacterial infections are diverse, widely spread, and highly detrimental, often resulting in substantial mortality rates that lead to significant economic losses in aquaculture. For example, common clinical signs of bacterial infections in fish include gill rot, septicemia, and enteritis, while crustaceans typically exhibit symptoms such as black gill disease and enteritis (Lafferty et al., 2015).

To eliminate pathogenic bacteria in the aquaculture process and prevent the occurrence of bacterial diseases in aquatic animals, various antibiotics, synthetic antimicrobial drugs, and other natural products (Ma et al., 2019) have been used. In aquaculture, antibiotics effectively combat bacterial pathogens, rapidly reducing bacterial populations and controlling disease outbreaks. However, their efficacy has led to misuse, resulting in residues in aquatic animals and environmental contamination, which poses risks to human health (Zhang et al., 2015). Furthermore, prolonged antibiotic use has fostered bacterial resistance through mechanisms such as enzyme production that modifies antibiotics or alterations in antibiotic targets (Nhung et al., 2024; Crofts et al., 2017; Blair et al., 2015; Allen et al., 2010), reducing their effectiveness. Therefore, identifying alternatives to antibiotics is crucial.

Certain organisms in nature utilize others as “prey” to acquire nutrients necessary for reproduction, such as phages (Cevallos-Urena et al., 2023) and *Bdellovibrio bacteriovorus* (Kongrueng et al., 2017). *Bdellovibrio and Bdellovibrio-like organisms* (BALOs) is a predatory bacterium that can either parasitize other bacteria or exist independently, leading to lysis of the host bacteria. Although it is smaller than typical bacteria, it can traverse bacterial filters such as phages (Thomashow and Rittenberg, 1985). Unlike phages, it is a bacterium capable of “consuming” a wide range of other bacteria. In addition, it was found that the addition of *Bdellovibrio* to aquaculture water significantly modified the microbial community structure on the gills of crucian carp, leading to enhanced survival rates (Zhang et al., 2023). Due to their unique predatory behavior and their ability to influence microbial community dynamics, they hold promise as beneficial agents for eliminating pathogenic microorganisms in aquaculture.

Despite numerous studies demonstrating the antibacterial potential of BALOs in mammals and aquatic organisms, its practical application is hindered by challenges such as suboptimal lysis efficiency. This study aimed to identify novel BALOs strains that can effectively treat aquaculture effluents and inhibit the growth of Gram-negative bacteria, such as *Vibrio*. In addition, a preliminary characterization of its fundamental properties and functions was conducted. The findings will significantly contribute to the identification of novel BALO strains and enhance the theoretical framework surrounding BALOs. In addition, these results will aid in the prevention and management of bacterial diseases in aquatic organisms, providing both practical and theoretical foundations for developing antibiotic alternatives in aquaculture.

## 2 Materials and methods

### 2.1 Enrichment culture of prey bacteria

*Escherichia coli* (a previously conserved strain in the key laboratory for healthy and safe aquaculture at South China Normal University) as prey bacteria was inoculated in sterilized lysogeny broth liquid medium and cultured on a shaker at 37°C under 180 rpm for 24 h. Then, the host cells were collected by centrifugation at 2400 × *g*, 4°C for 15 min. Then, the resulting bacterial pellet was gently washed twice and re-suspended to reach a final concentration of 10^10^ colony-forming units (CFU)/mL with sterile 0.9% saline solution. This bacterial suspension was reserved at 4°C for subsequent experiments.

### 2.2 Sample collection, treatment, and isolation of *Bdellovibrio*-like organisms (BALOs)

Water sampling was conducted in a freshwater fish pond at South China Normal University. A total of 500 mL water samples were collected at a depth of 10 cm from the surface of the fish pond using a water harvester. The samples were allowed to settle on ice for 30 min to facilitate the sedimentation of debris and larger particles. Subsequently, the supernatant was centrifuged for 5 min at 4°C and 100 × *g* for purification. A mixture of 50 mL of the centrifuged supernatant and 1 mL of *E. coli* (initial concentration 10^10^ CFU/mL) was prepared in a sterilized triangular flask and incubated in an oscillator at 30°C and 180 rpm until clarification occurred. The lysate was then placed on ice for 30 min to precipitate larger particles, followed by centrifugation at 400 × *g* for 10 min at 4°C to eliminate larger bacterial organisms, thereby obtaining the lysate supernatant.

The BALOs was isolated using the double-layer agar plating technique (Stolp and Starr, 1963): 0.5 mL of the treated lytic supernatant and 0.2 mL of *E. coli* (with an initial concentration of 10^10^ CFU/mL) were mixed into 5 mL of 0.6% agar medium containing CaCl_2_ (at a final concentration of 3 mM). This mixture was then poured onto pre-solidified 1.5% agar medium. A negative control was established by mixing 0.5 mL of sterile tap water with 0.2 mL of *E. coli* in 5 mL of the same 0.6% agar medium containing CaCl_2_ to validate the specificity of plaque formation. The plates were then incubated in a 30°C environment for 7 days (d) after the top agar solidified at room temperature, with observations made every 12 h.

After the initial plate culture for 48 h, an individual gradually enlarging plaque was carefully transferred into a tube containing 5 mL of sterilized tap water and 0.05 mL of 0.3 M CaCl_2_ solution. This solution was then shaken on a shaker at a speed of 180 rpm and a temperature of 30°C for a total of 8 h. This soaking solution was purified using the double-layer agar plates. This same procedure was repeated until a plaque with uniform size and transparency was obtained, indicating that BALOs had been successfully purified.

### 2.3 Microscopic observation of BALOs

The fresh BALOs lysate underwent a centrifugation at 400 × *g* and 4°C for 10 min. A droplet of supernatant was placed on a microscope slide and subjected to Gram staining utilizing the Biosharp kit, for subsequent observation under a standard optical microscope.

To enhance the morphological assessment of BALOs, a supernatant drop was placed on a 300-mesh copper net, sustained for 10, and subsequently sucked dry on filter paper. The copper net was then immersed in a 2% phosphotungstic acid stain for 90 s, clipped, and dried. In addition, a drop of sample supernatant was mixed with a drop of stain, and then, the stain on the copper net was washed off with the mixed solution for four times. The copper net was air-dried and studied under a transmission electron microscope (TEM).

### 2.4 Analysis of partial 16S rRNA and hit gene of BALOs

Genomic DNA extraction was performed with the TaKaRa MiniBEST Universal Genomic DNA Extraction Kit Ver.5.0, following the manufacturer’s protocol. The concentration of the genomic DNA was quantified with a NanoDrop ONE spectrophotometer (Thermo Fisher Scientific, America) as well as checked on 1% agarose gel electrophoresis. The extracted DNA is preserved at −20°C.

According to the specific primer sequence designed by Edouard and Robert (2020), the following sequence was synthesized by IGEbio company (Table 1). For phylogenetic analysis, the 16S rRNA gene was selectively amplified by PCR using BALOs-specific primers in a total reaction system of 50 uL: primers (10 μM) 1 μL each, 2 × rTaq premix enzyme 25 μL, DNA template 2 μL, sterile distilled water for volume completion. The optimized thermal cycling parameters were 95°C for 3 min to activate the polymerase, followed by 33 cycles of 98°C for 10 s, 55°C for 30 s, and 72°C for 1 min, and the final extension step at 72°C for 5 min. PCR products (~800 bp) displayed successful amplification via 1% agarose electrophoresis, subsequently purified and dispatched to IGEbio company for sequencing. The measured *16S rRNA* partial sequences were compared with those in NCBI database. Multiple sequence alignments were performed using the Cluster X program. A phylogenetic tree was constructed through the Minimum Evolution (*p*-distance model) using MEGA 11 software with a bootstrap analysis of 1, 000 replicates.

**Table 1:** Related primers.

No.	Primers	Sequence (5′ → 3′)
1	Bacteria-normal-*F* (63)	CAGGCCTAACACATGCAAGTC
2	BALOs-R (842)	CGWCACTGAAGGGGTCAA
3	BALOs-Bacteriovorus-R (435)	ATTCCCCTGCGACAGAG
4	*hit*-F	TCTAGACAGATGGGATTACTG
5	*hit*-R	GAATTCTGGCATCAACAGC

*Bdellovibrio bacteriovorus*-specific primers were used to ensure genus of the purified strain. PCR was performed at a total volume of 50 uL comprised of 1 uL gDNA, 25 uL 2 × rTaq premix enzyme, 1 uL of each primer, and sterile distilled water for volume completion. The optimized thermal cycling parameters were 95°C for 3 min to activate the polymerase, followed by 33 cycles of 98°C for 10 s, 55°C for 30 s, and 72°C for 30 s, and the final extension step at 72°C for 5 min. PCR products (~400 bp) displayed successful amplification via 1% agarose electrophoresis.

According to the *hit* gene primer sequence designed by Oyedara et al. (2016), the following sequence was synthesized by IGEbio company (Table 1). The PCR system used was the same as that described above, with a total volume of 50 μL. The optimized thermal cycling parameters were 95°C for 3 min to activate the polymerase, followed by 28 cycles of 98°C for 10 s, 55°C for 30 s, and 72°C for 1 min, and the final extension step at 72°C for 5 min. PCR products (~950 bp) displayed successful amplification via 1% agarose electrophoresis.

### 2.5 Biochemical index identification

Samples were submitted to Shanghai Fuda Detection Technology Group Co., Ltd. for biochemical identification, scrutinizing oxidase, contact enzyme, arginine decarboxylase, gelatin liquefaction, indole test, citrate, nitrate reduction, urease, hydrogen sulfide, and methyl red tests.

### 2.6 Investigate the lysis ability of BALOs to diverse host

*Escherichia coli*, *Aeromonas hydrophila*, *Vibrio alginolyticus*, *Vibrio Parahaemolyticus*, or *Edwardsiella tarda* (initial concentration of 10^10^ CFU/mL and final concentration of 10^8^ CFU/mL) were co-cultured with *bacteria by B. bacteriovorus FWA* (initial concentration of 2 × 10^5^ PFU/mL and final concentration of 10^4^ PFU/mL) on the double-layer agar plates at 30°C for 7 d, with plaque observations performed every 24 h. Meanwhile, the previously mentioned pathogenic bacteria were individually cultured on double-agar plates without BALOs as a negative control.

### 2.7 Consequences of different concentrations of *Escherichia coli* on bacteria by BALOs growth

The prepared washed *E. coli* suspensions (10^5^, 10^6^, 10^7^, 10^8^, or 10^9^ CFU/mL) were, respectively, co-cultured with BALOs (at a final concentration of 2 × 10^4^ PFU/mL) at 30°C for 4 days under 180 rpm, with three replicates per group. The BALOs-free groups on different concentrations of *E. coli* served as negative control. The BALOs count variations (PFU/mL) were monitored at 24-h intervals.

### 2.8 Effects of oxygen availability on the growth and predation of BALOs

The assessment of oxygen’s influence on the growth and predation of BALOs was conducted via co-culturing washed *E. coli* suspensions (at a final concentration of 10^8^ CFU/mL) and BALOs (at a final concentration of 10^4^ PFU/mL) at 30°C under 180 rpm. Vaseline was applied to the anaerobic group to maintain anaerobic conditions, whereas the aerobic group remained untreated. Three replicates were performed for each treatment. The BALOs-free groups consisted solely of the *E. coli* suspension, which were incubated in various oxygen environments as negative control. BALOs quantitative changes (PFU/mL) were monitored at 24-h intervals, and lysis capacity (OD600) was monitored at 24-h intervals.

### 2.9 Effects of pH and temperature on the growth and predation of BALOs

The effects of pH on the growth and predation of BALOs were investigated by co-culturing with *E. coli* (at a final concentration of 10^8^ CFU/mL) and BALOs (at a final concentration of 10^4^ PFU/mL) at 30°C under 180 rpm under diverse pH conditions (pH 5, 6, 6.5, 7, 7.5, 8, 9, or 10). For temperature influence, *E. coli* and BALOs were co-cultured at pH 7.5 and 180 rpm but varied in temperature (20°C, 25°C, 30°C, 35°C, or 40°C). Each experimental group was replicated three times. The BALOs-free groups consisted solely of the *E. coli* suspension, which were incubated in different pH or temperature conditions as negative control. BALOs quantitative changes (PFU/mL) were monitored at 24-h intervals, and lysis capacity (OD600) was monitored at 24-h intervals.

### 2.10 Effects of Ca^2+^ and Na^+^ on the growth and predation of BALOs

Different concentrations of CaCl_2_ (0 mM, 3 mM, 5 mM, 8 mM, 10 mM, 15 mM, 20 mM, 25 mM, or 30 mM), *E. coli* (at a final concentration of 10^8^ CFU/mL), and BALOs (at a final concentration of 10^4^ PFU/mL) were added to a culture system and cultivated at 30°C under 180 rpm to systematically probe the impact of Ca^2+^ on bacterial growth and predation of BALOs. Each experiment was repeated three times. BALOs quantitative changes (PFU/mL) were monitored at 24-h intervals, and lysis capacity (OD600) was monitored at 24-h intervals. The Na^+^ effect was investigated at 30°C under 180 rpm with different concentrations of NaCl (0, 0.5, 1, 1.5%, or 2%) in the same way as mentioned for different concentrations of Ca^2+^ effect. The BALOs-free groups only included the *E. coli* suspension, which were incubated in varying concentrations of Ca^2+^ or Na^+^ as a negative control.

### 2.11 Impact of *Bdellovibrio bacteriovorus FWA* application on aquaculture effluent from freshwater ponds

Twelve 10 L tanks were utilized for the untreated aquaculture wastewater, with each tank containing 6 L of aquaculture effluent. The water temperature was controlled between 27 and 29°C, and air stones were used to provide oxygenation. Three experimental groups were established: the 10^3^ PFU/mL *B. bacteriovorus FWA* group, the 10^2^ PFU/mL *B. bacteriovorus FWA* group, and the 10^1^ PFU/mL *B. bacteriovorus FWA* group, alongside a negative control group lacking *B. bacteriovorus FWA*. The experiment lasted for 7 days.

To assess the variations in total bacterial counts between the experimental and control groups, water samples were collected at 0, 24, 48, 72, 96, 120, 144, and 168 hpc. The samples were diluted, and the total bacterial number of water samples was calculated by dilution plate counting. To assess variations in ammonia nitrogen and nitrite nitrogen levels in water samples, samples were collected every 12 hpc, spanning a duration of 0 to 168 h. Ammonia nitrogen was quantified using a photometric technique (Okumura et al., 2005), while nitrite nitrogen was analyzed via the N-(1-naphthyl)-ethylenediamine photometric method (Helaleh and Korenaga, 2001). The initial concentrations of ammonia nitrogen and nitrite nitrogen in the water were 0.10 ± 0.02 mg/L and 0.01 ± 0.00 mg/L, respectively.

### 2.12 Statistical analyses

All experiments were carried out in triplicate, and each test was repeated three times. A *p*-value of < 0.05 was considered as statistically significant. The results were presented as the mean ± standard error of mean (SEM). Statistical analyses were performed with GraphPad Prism V9 and IBM SPSS Statistics 26.

## 3 Results

### 3.1 Isolation of a BALOs from aquatic freshwater

**Figure 1:**
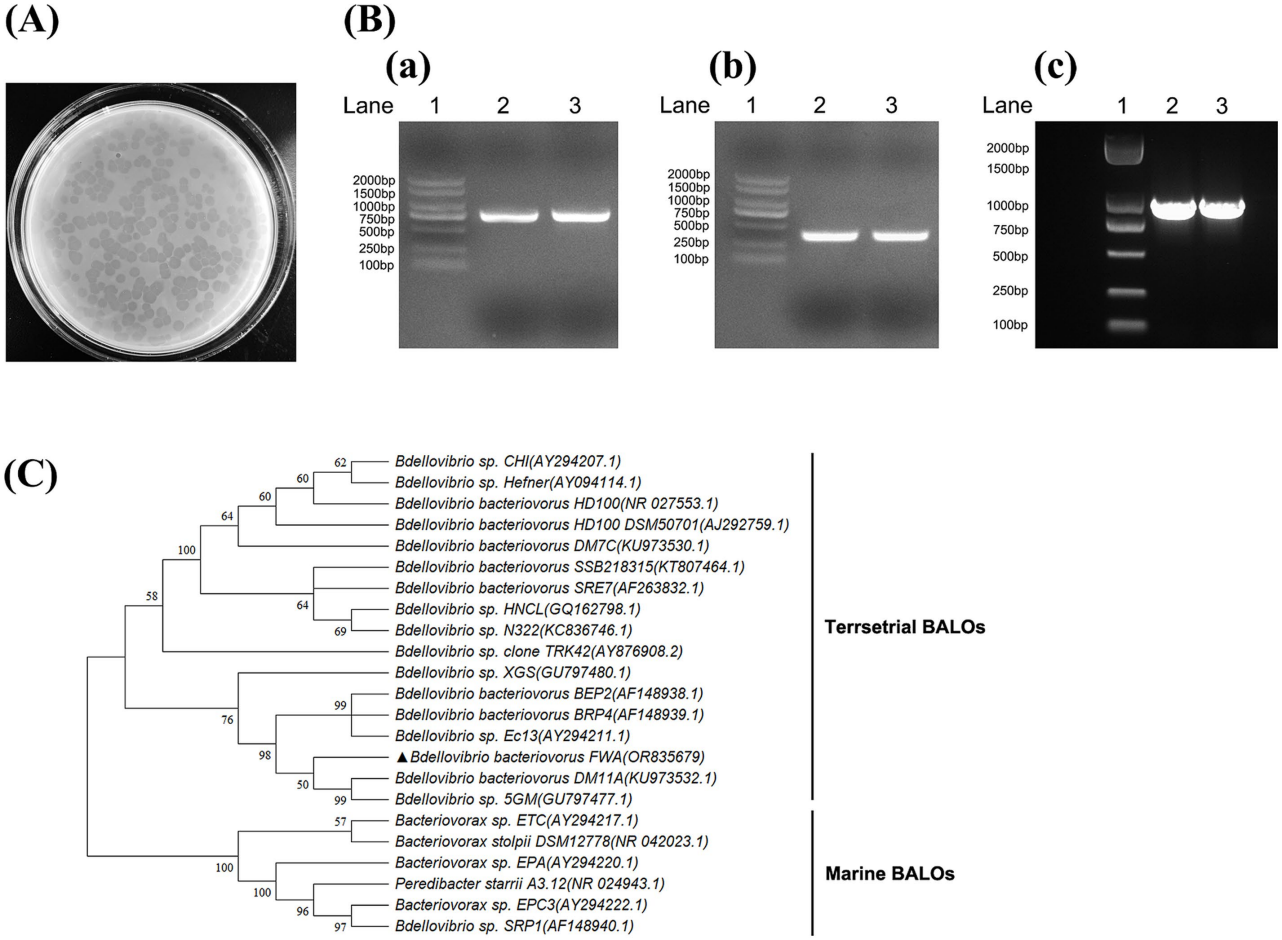
Isolation and identification of *Bdellovibrio bacteriovorus*. **(A)** Plaques formed by *B. bacteriovorus FWA*. After 96 h of incubation on double-layer agar culture containing *E. coli*, *B. bacteriovorus FWA* formed circular plaques with a diameter of 0.5–0.7 cm. **(B)** Amplification of 16S rRNA partial fragment and *hit* gene fragment of *B. bacteriovorus FWA*. (a) Amplification of the 16S rRNA partial gene sequence of *Bdellovibrio* (~800 bp). (b) Amplification of the 16S rRNA partial gene sequence of *Bdellovibrio bacteriovorus* (~400 bp). (c) Amplification of the *hit* gene sequence of *Bdellovibrio.* Lane 1 is 2, 000 bp marker, and lanes 2 and 3 are the amplification results of *B. bacteriovorus FWA*. **(C)** Phylogenetic tree of partial 16S rRNA gene sequences of *B. bacteriovorus FWA*. The Minimum Evolution phylogenetic tree was constructed through MEGA 11 software by using p-distance method. The tree was generated using bootstrap sampling, which was carried out 1,000 times. *B. bacteriovorus FWA* is marked with triangles.

The water samples from freshwater fish ponds were initially enriched for BALOs in the presence of *E. coli* to increase predator density to sufficiently large to be observed by subsequent plaque assays. After 3–5 d of incubation on the double-layer agar plates, lytic plaques that were transparent and round with diameters ranging from 0.5 to 0.7 cm were observed in 96 h (Figure 1A). These plaques expanded after longer incubation (after 4 d of incubation), which eventually covered almost the entire lawn. These results suggested that predators were motile within the soft agar, which differentiated them from the types of plaques typically associated with bacteriophages.

### 3.2 The BALOs isolate was identified as *Bdellovibrio bacteriovorus*

The target *16S rRNA* fragment and *hit* genes of BALOs were successfully amplified from isolation using the above primers, with amplicon sizes matching expectations (Figure 1B). Alignment of the *16S rDNA* gene sequences isolated from GenBank database indicated that the predator was a member of the *Bdellovibrio* genus, with 98.89% identity to *Bdellovibrio* sp. *Ec13* (Figure 1C). Therefore, the isolated predatory bacterium was suggested to be a strain of *B. bacteriovorus*, hereinafter referred to as *B. bacteriovorus FWA* (NCBI No. OR835679), for *Bdellovibrio bacteriovorus* strain fishpond water-A.

**Figure 2:**
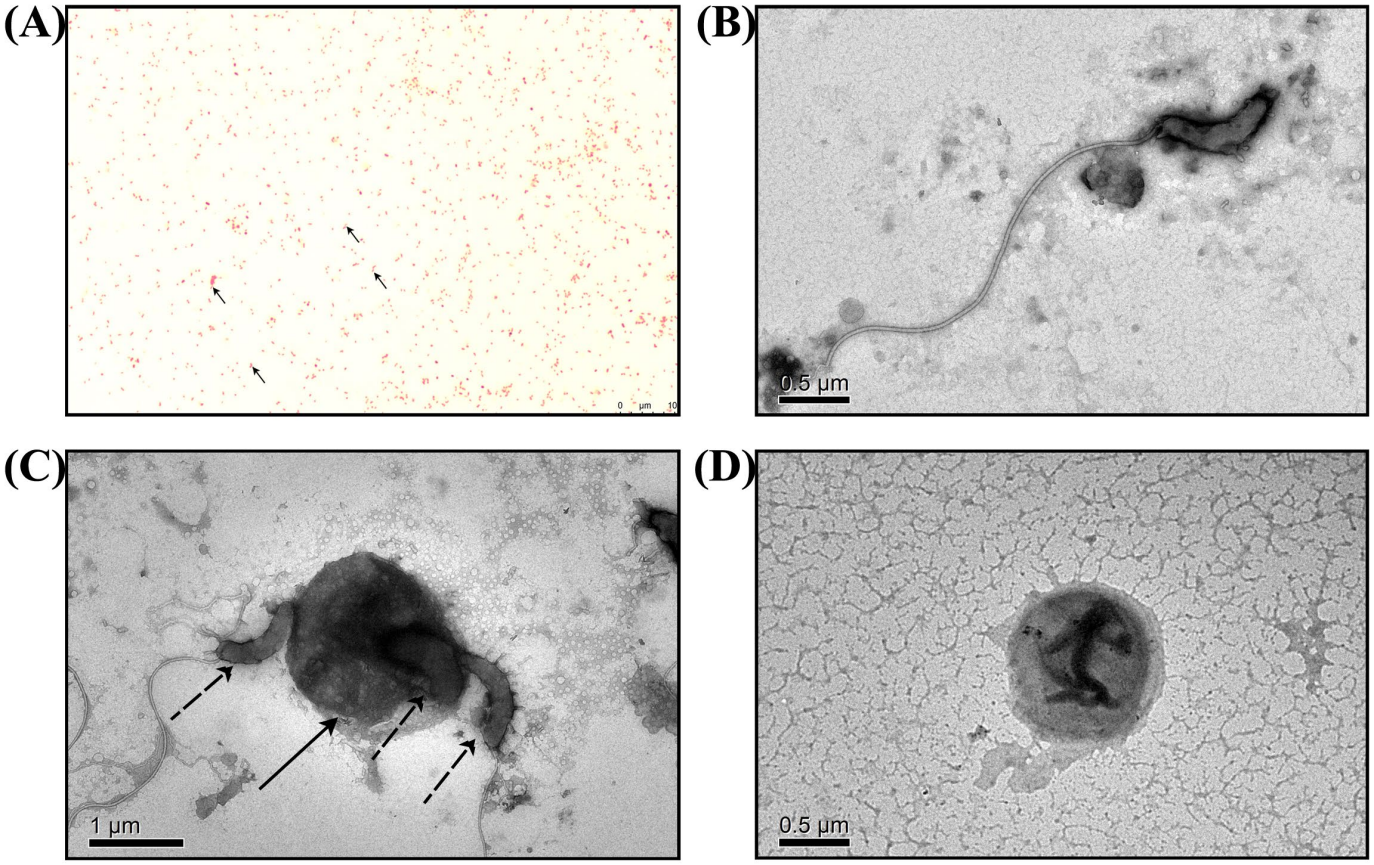
Microscopic observation of *B. bacteriovorus FWA*. **(A)** Following Gram staining and observation under an oil microscope (1000×), it was determined that *B. bacteriovorus FWA* are Gram-negative bacteria. **(B)** The state of *B. bacteriovorus FWA*-free attack in TEM (6000×). **(C)** The state of *B. bacteriovorus FWA* invading host bacteria in TEM (4000×); solid arrow is the host bacterium, and dashed arrows are *B. bacteriovorus FWA*. **(D)** The state of *B. bacteriovorus FWA* bdelloplast in TEM (6000×).

*BALO* co-cultures with *E. coli* were subjected to Gram staining for observation (Figure 2A). In addition to *E. coli*, which with short rod-shaped and 0.5 mm wide approximately, there were also bar bacteria that 0.4–0.5 μm wide and 0.8–0.9 μm long approximately. Further TEM observations revealed that the bacteria had long single flagella at one end. These results suggested that *Bdellovibrio* sp. was present in the co-culture (Figures 2B–D).

Analysis by FUDA ANALYTICAL TESTING GROUP (China) showed that the oxidase, citrate, and nitrate reduction tests of *B. bacteriovorus FWA* were negative, and the contact enzyme, arginine decarboxylase, gelatine, indole, urease, hydrogen sulfide, and methylation tests of *B. bacteriovorus FWA* were positive (Table 2).

**Table 2:** Results of biochemical tests.

No.	Items	Results
1	Oxidase	−
2	Contact enzyme	+
3	Arginine decarboxylase	+
4	Gelatine	+
5	Indole	+
6	Citrate	−
7	Nitrate reduction	−
8	Urease	+
9	Hydrogen sulfide	+
10	Methyl red	+

### 3.3 *Bdellovibrio bacteriovorus FWA* exhibited host cell density-dependent growth kinetics

**Figure 3:**
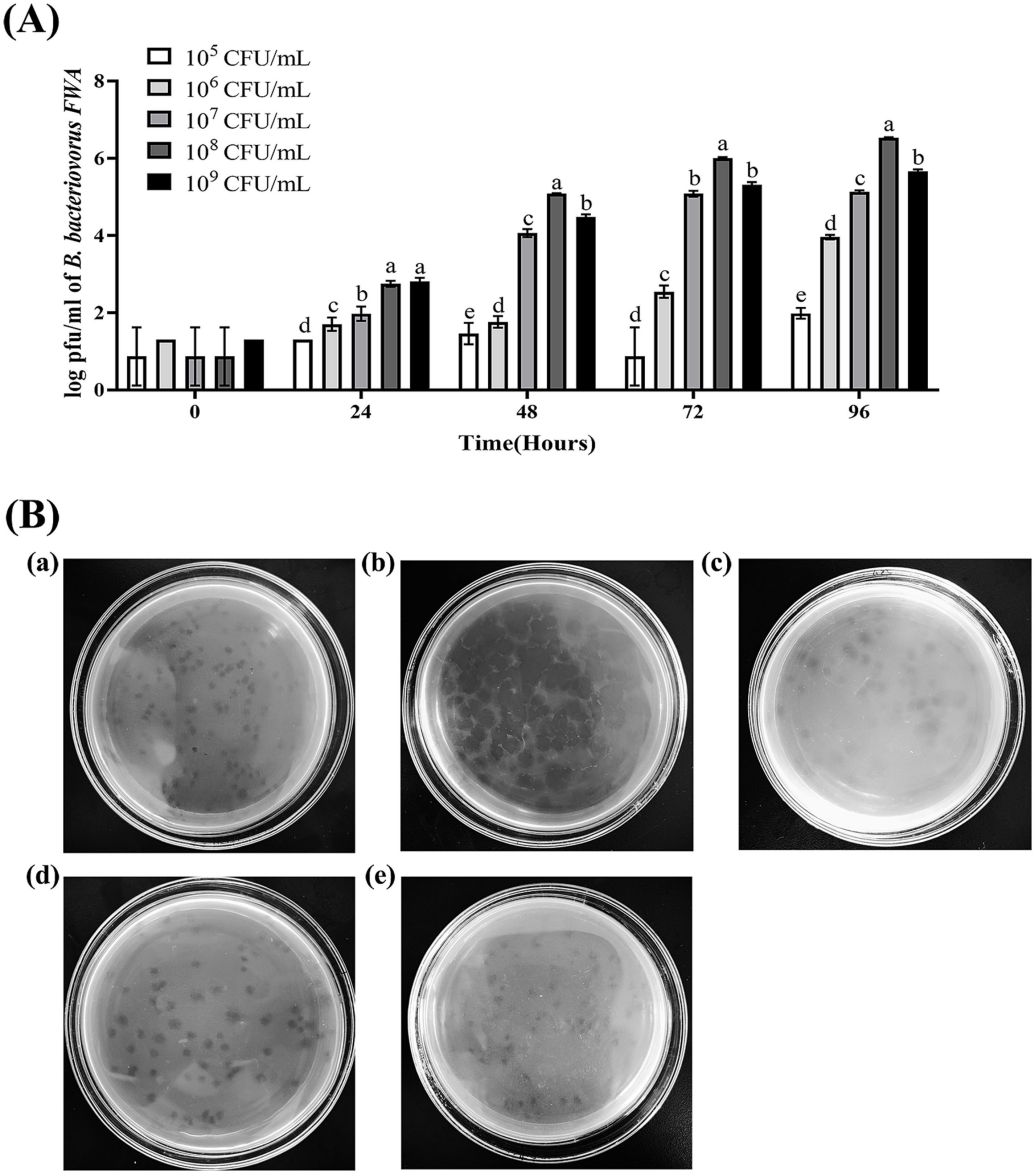
Effect on *B. bacteriovorus FWA* growth and cleavage of different host bacterial concentrations and different host bacteria. **(A)** Growth of strain *B. bacteriovorus FWA* at different host bacteria (*E. coli*) concentrations, *B. bacteriovorus FWA* was cultivated through the incorporation of varying concentrations of host bacteria (*E. coli*) in the liquid medium, followed by sampling at 0, 24, 48, 72, and 96 h. These samples were then carried out prior to enumeration through the double-agar plates. **(B)** Phage spots formed by *B. bacteriovorus FWA* on different host bacteria: (a) *V. alginolyticus*, (b) *E. coli*, (c) *V. parahaemolyticus*, (d) *A. hydrophila*, and (e) *E. tarda*. The results are based on three independent experiments and the population of bacteria (log/mL) as the mean values ± SEM.

Since *Bdellovibrio* obtains nutrients from parasitic host bacteria and cleaves the host bacteria to achieve value appreciation, the plaques or OD600 of the co-culture bacteria solution can roughly reflect the change in its titer. In this study, *E. coli* was used as the host bacteria of *B. bacteriovorus FWA* and the initial concentration of *B. bacteriovorus FWA* was 10^4^ CFU/mL. When the bait bacteria concentration was 10^8^ CFU/mL, the number of *B. bacteriovorus FWA* released progeny was much higher than other bait bacteria concentrations, and the 48 h post-co-culture (hpc) and 96 hpc the number of plaques reached 5.08 ± 0.01 (log, PFU/mL) and 6.54 ± 0.01 (log, PFU/mL), respectively. At 96 hpc, the *B. bacteriovorus FWA* reproduction efficiency decreased for groups that host concentrations of 10^7^ CFU/mL or 10^9^ CFU/mL, compared with 10^8^ CFU/mL group. For the former two groups, their plaque numbers were 5.13 ± 0.02 (log) and 5.65 ± 0.03 (log), respectively. In the groups with concentrations of 10^5^ CFU/mL and 10^6^ CFU/mL, the reproduction efficiencies of *B. bacteriovorus FWA* both were relatively low. At 96 hpc, their plaque numbers were 4.55 ± 0.08 (log, PFU/mL) and 2.58 ± 0.03 (log, PFU/mL), respectively. By calculation, the optimal ratio of *B. bacteriovorus FWA* to host bacteria was approximately 1:10000 (Figure 3A).

### 3.4 *Bdellovibrio bacteriovorus FWA* can prey upon several difference host bacteria

*Escherichia coli*, *A. hydrophila*, *V. alginolyticus*, *E. tarda*, and *V. parahaemolyticus* were tested for their susceptibility to predation by *B. bacteriovorus FWA*. In the double-layer agar cultures with *E. coli*, *A. hydrophila*, or *E. tarda* as prey, plaques appeared 36 hours post inoculation, yielding titers of 3.30×10^4^ PFU/mL, 3.31×10^4^ PFU/mL, and 2.06×10^4^ PFU/mL, respectively. Conversely, in the cultures with *V. alginolyticus* and *V. parahaemolyticus*, plaques were observed 48 hours after inoculation of *B. bacteriovorus FWA*, resulting in phage titers of 2.80×10^4^ PFU/mL and 2.11×10^4^ PFU/mL, respectively (Table 3). 96 h post-*B. bacteriovorus FWA* inoculation, diameter of plaques for double-layer agar culture with *E. coli*, *A. hydrophila*, *V. alginolyticus*, *E. tarda*, and *V. parahaemolyticus* prey was 0.35–0.5 cm, 0.25–0.35 cm, 0.3–0.5 cm, 0.25–0.35 cm, and 0.25–0.4 cm, respectively. In addition, for double-layer agar culture with *E. coli* or *A. hydrophila* prey, edge of the plaques was clear, and for double-layer agar culture with *V. alginolyticus*, *E. tarda*, or *V. parahaemolyticus* prey, edge of the plaques was foggy (Figure 3B).

**Table 3:** Cleavage of different host bacteria by *B. bacteriovorus FWA.*

Host bacteria	Time of occurrence	Quantity (PFU/mL)	Patchy phenomenon	Phage size (96 h)
*Vibrio alginolyticus*	48 h	2.8 × 10^4^	Distinct	0.1–0.3 cm
*Escherichia coli*	36 h	3.30 × 10^4^	Distinct	0.45–0.65 cm
*Vibrio parahaemolyticus*	48 h	2.11 × 10^4^	Vague	0.25–0.4 cm
*Aeromonas hydrophila*	36 h	3.31 × 10^4^	Distinct	0.25–0.35 cm
*Edwardsiella*	36 h	2.06 × 10^4^	Vague	0.25–0.35 cm

### 3.5 Oxygen supply improves the proliferation vitality of *Bdellovibrio bacteriovorus FWA*

**Figure 4:**
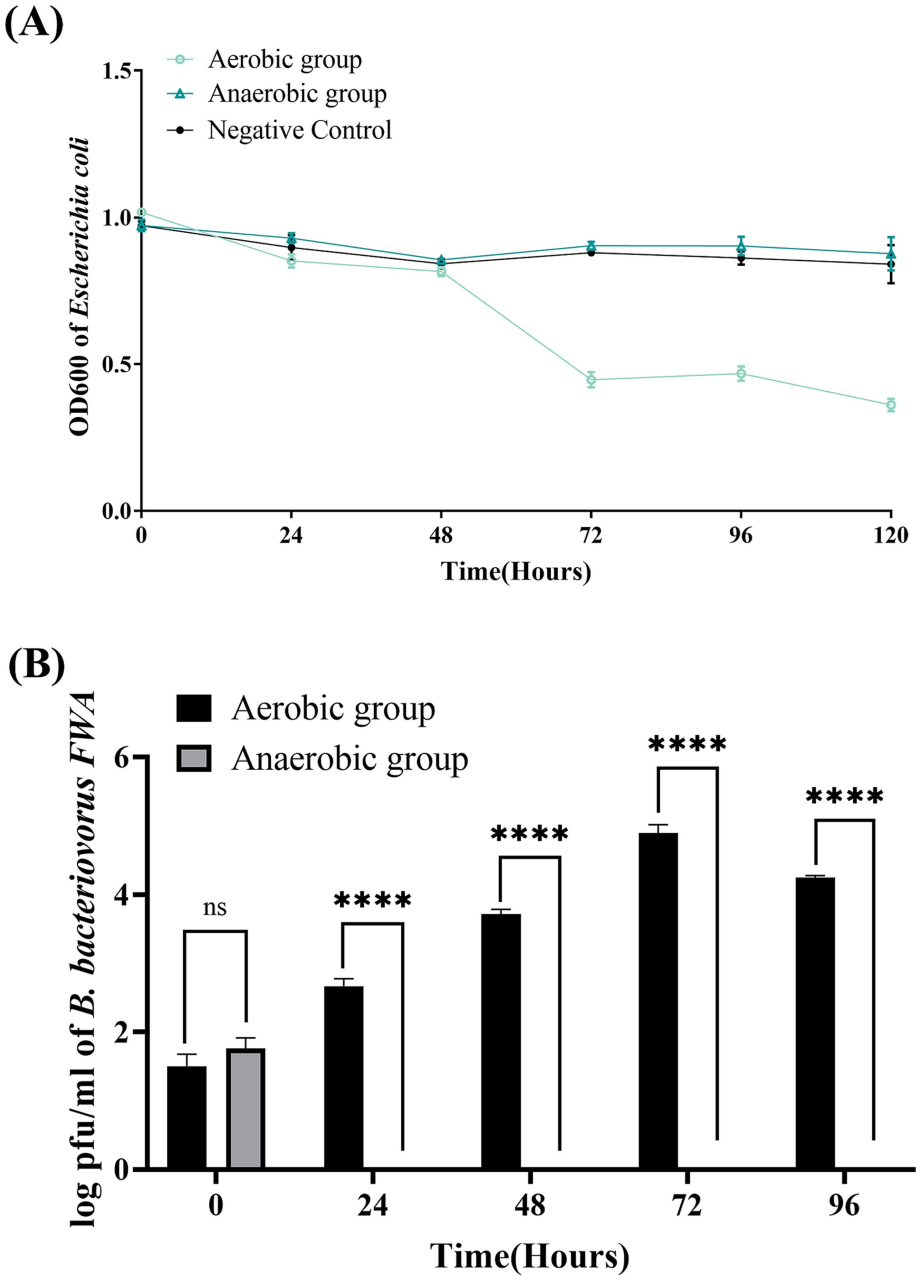
Effect of oxygen on the growth and cleavage of *B. bacteriovorus FWA*. **(A)** The predation of *B. bacteriovorus FWA* on the host bacteria (*E. coli*) in liquid media under varying oxygen conditions is evaluated by changes in OD600. All experiments are performed in triplicate, significance analysis uses one-way analysis of variance (ANOVA), *p* < 0.05, and the results are the mean value ± SEM. **(B)** *B. bacteriovorus FWA* growth was examined under varying oxygen conditions, samples were collected at 0, 24, 48, 72, and 96 h from the co-culture of *B. bacteriovorus FWA* with *E. coli*, and *B. bacteriovorus FWA* enumeration was performed via double-agar plate method. All experiments are performed in triplicate, significance analysis uses two-way ANOVA, **** *p* < 0.0001, and error bars represent standard error of mean.

OD600 of bacterial solution decreased significantly in the aerobic group during 48–72 hpc, while OD600 bacterial solution in the anaerobic group remained almost unchanged during the experimental period. This result confirmed that oxygen supply has a significant effect on promoting the lysis capacity of *B. bacteriovorus FWA*. At 72 hpc, the bacterial solution OD600 of aerobic group decreased by 0.57 ± 0.02 (*p* < 0.05), while the bacterial solution OD600 of anaerobic group decreased by 0.07 ± 0.00 (*p* < 0.05) (Figure 4A). For the aerobic group, the plaque assay showed that *B. bacteriovorus FWA* gradually increased during the experiment. Plaque numbers peaked at 72 hpc, which was 4.89 ± 0.07 (log), and the number of *B. bacteriovorus FWA* decreased from 96 hpc. For the anaerobic group, the number of plaques continued to decrease, and after 24 hpc, no plaques appeared on double-layer agar culture (Figure 4B).

### 3.6 Bacterial solution pH affected *Bdellovibrio bacteriovorus FWA* growth

**Figure 5:**
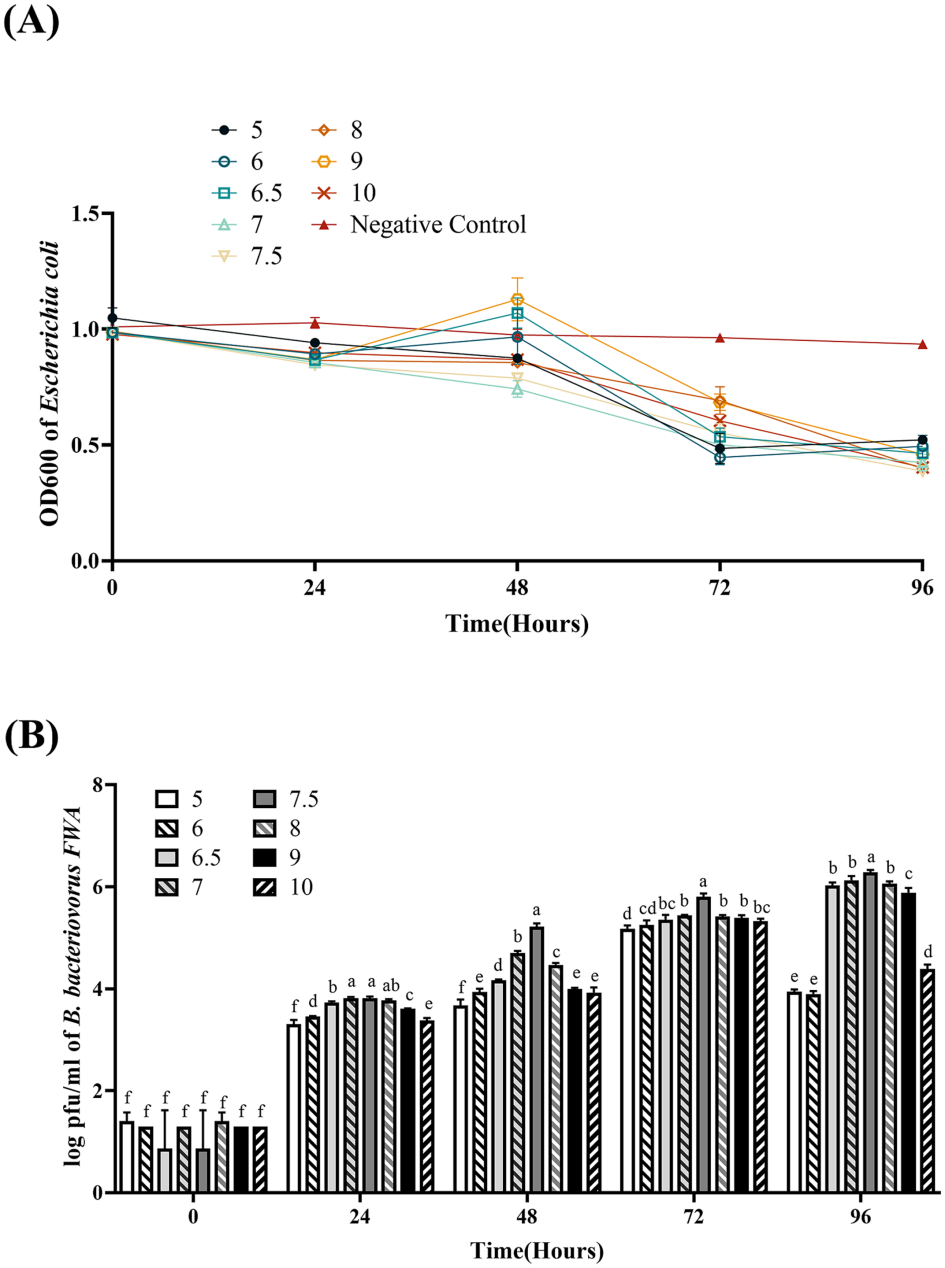
Effect of pH on the growth and cleavage of *B. bacteriovorus FWA*. **(A)** The predation of *B. bacteriovorus FWA* on the host bacteria (*E. coli*) in liquid media under different pH environments is evaluated by changes in OD600, **(B)** *B. bacteriovorus FWA* growth was examined under different pH conditions, samples were collected at 0, 24, 48, 72, and 96 h from the co-culture of *B. bacteriovorus FWA* with *E. coli,* and *B. bacteriovorus FWA* enumeration was performed via double-agar plate method. All experiments are performed in triplicate, significance analysis uses one-way ANOVA, *p* < 0.05, and the results are the mean value ± SEM.

*Bdellovibrio bacteriovorus FWA* could grow well at pH 5.0–10.0. At pH 7.0 and pH 7.5, the co-culture bacterial solution OD600 began to decrease from 48 hpc, which *B. bacteriovorus FWA* grew faster than in other pH environments. *B. bacteriovorus FWA* grew more slowly at pH 5.0 and pH 10.0, and the OD600 bacterial solution began to decline after 72 hpc. At 96 hpc, the bacterial solution OD600 showed the largest reduction at pH 7.0–8.0, with pH 7.5 and 8.0 being 0.60 ± 0.01 and 0.59 ± 0.01, respectively (Figure 5A). Similarly, the plaque assay confirmed that *B. bacteriovorus FWA* grow well at pH 7.0–8.0. At pH 7.5, the number of plaques at 48 hpc and 96 hpc was 5.22 ± 0.04 (log) and 6.28 ± 0.03 (log), respectively (Figure 5B).

### 3.7 Bacterial solution temperature affected *Bdellovibrio bacteriovorus FWA* growth

**Figure 6:**
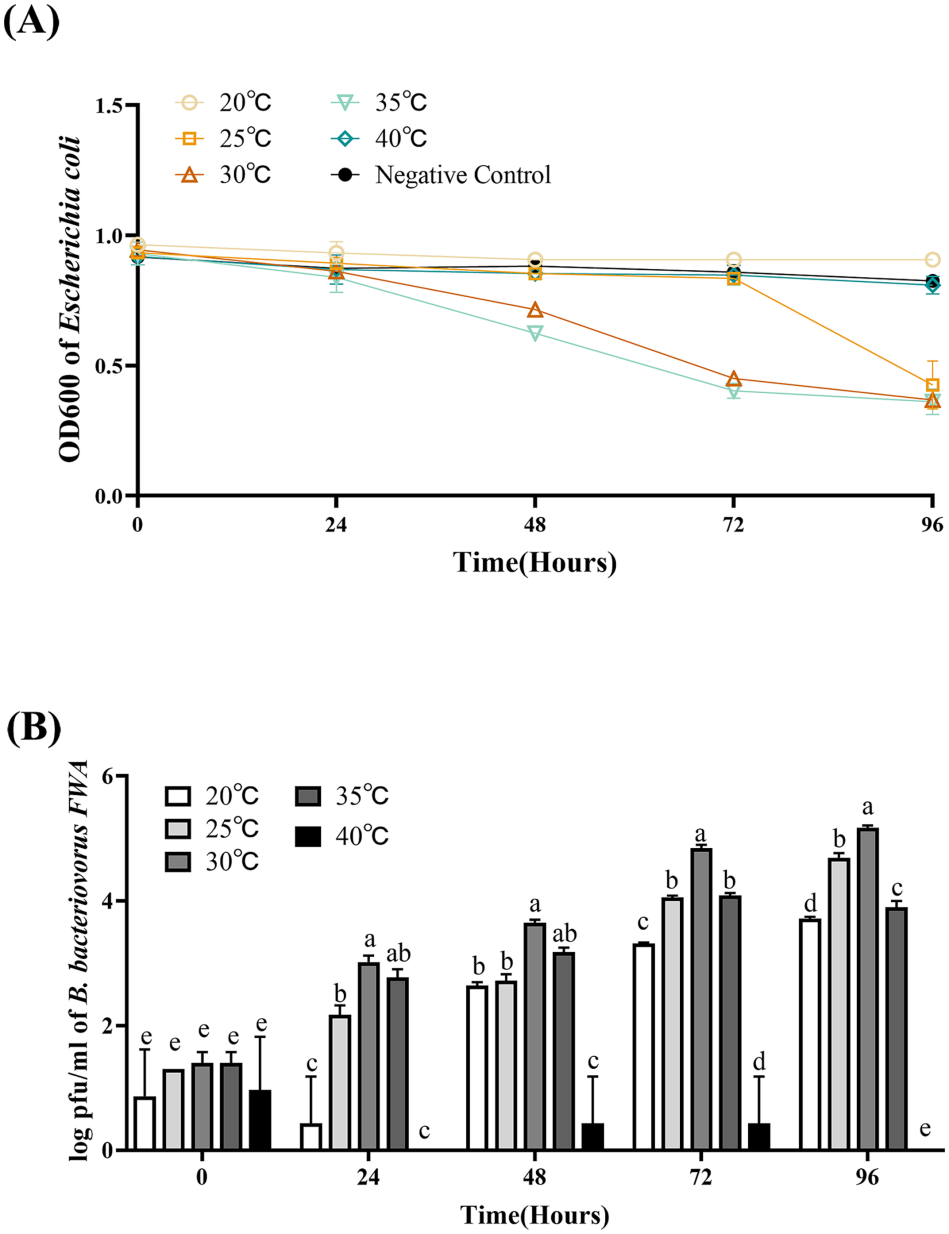
Effect of different temperatures on the growth and cleavage of *B. bacteriovorus FWA*. **(A)** The predation of *B. bacteriovorus FWA* on the host bacteria (*E. coli*) in liquid media under different temperature is evaluated by changes in OD600, **(B)** *B. bacteriovorus FWA* growth was examined under different temperature, samples were collected at 0, 24, 48, 72, and 96 h from the co-culture of *B. bacteriovorus FWA* with *E. coli,* and *B. bacteriovorus FWA* enumeration was performed via double-agar plate method. All experiments are performed in triplicate, significance analysis uses one-way ANOVA, *p* < 0.05, and the results are the mean value ± SEM.

The optimal temperature range for *B. bacteriovorus FWA* growth is 30°C–35°C. Bacterial solution OD600 of the 30°C group and 35°C culture group began to decrease after 36 hpc. For the 30°C group and 35°C group, at 72 hpc, the bacterial solution OD600 begun to decrease, which was 0.53 ± 0.02 and 0.50 ± 0.02, respectively. For the 25°C group, *B. bacteriovorus FWA* showed a little weaker grew activity, which lysed host bacteria massively after 84 hpc–96 hpc. For the 20°C group and 40°C group, bacterial solution OD600 was almost unchanged in the duration of the experiment (Figure 6A).

The results of plaques assay showed that at 30°C, *B. bacteriovorus FWA* had the best growth and reproduction ability. Over the 24 hpc–96 hpc period, the overall trend of plaque numbers was higher than in other temperature groups. For the 30°C group, at 48 hpc, 72 hpc, and 96 hpc, the number of plaques was 3.6466 ± 0.03 (log), 4.84 ± 0.03 (log), and 5.17 ± 0.03 (log), respectively. At 35°C, *B. bacteriovorus FWA* had similar growth and reproduction ability to that at 30°C, and the number of plaques at 72 hpc and 96 hpc was 4.84 ± 0.03 (log) and 4.09 ± 0.02 (log), respectively. At 20°C and 25°C, *B. bacteriovorus FWA* growth and reproduction efficiency decreased, and the progeny of *B. bacteriovorus FWA* was released slowly, and the plaque numbers at 48 hpc and 96 hpc were 2.64 ± 0.03 (log) and 2.72 ± 0.06 (log), respectively (Figure 6B).

### 3.8 *Ca*^*2*+^ concentration has an influence on *Bdellovibrio bacteriovorus FWA* growth

**Figure 7:**
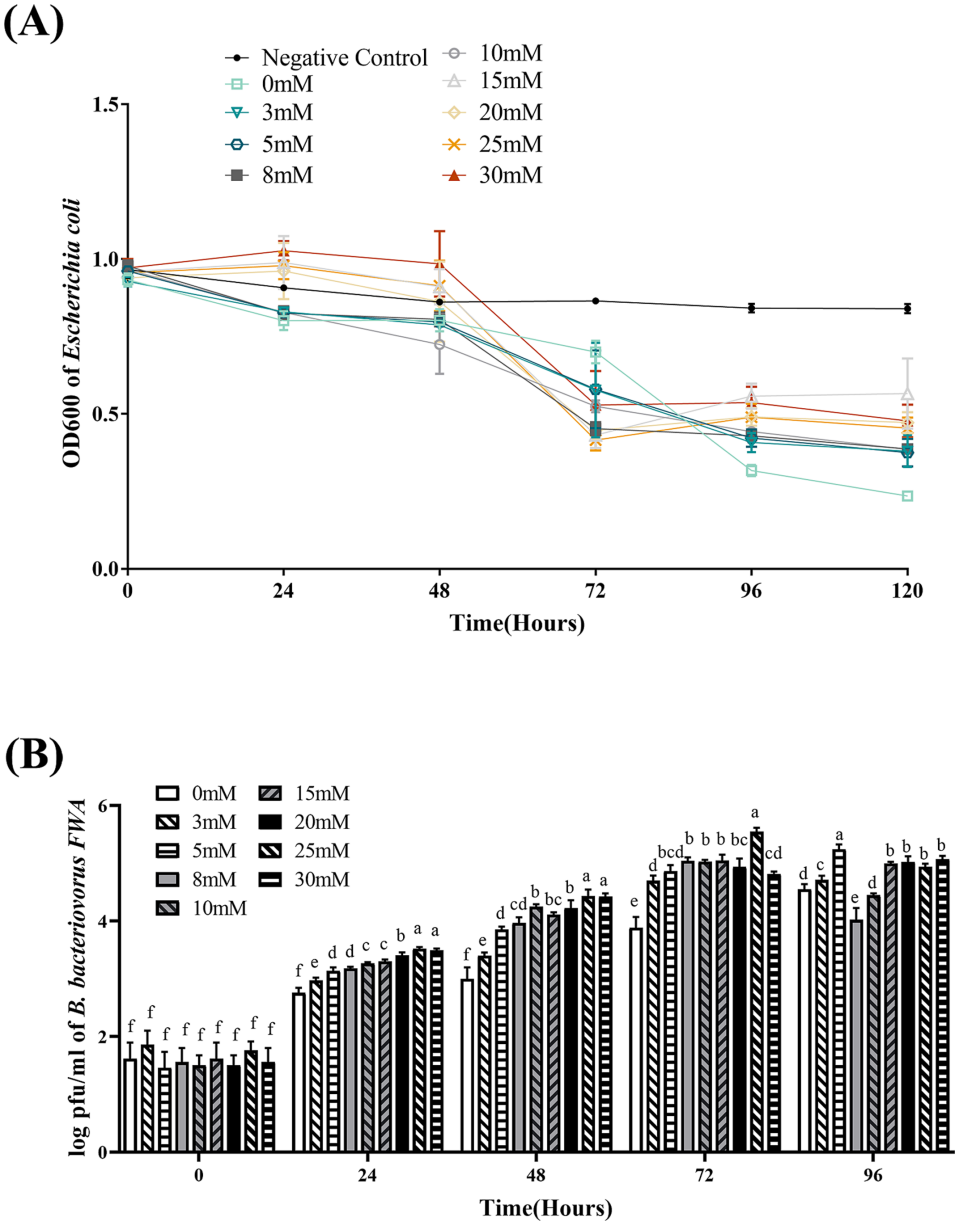
Effect of different Ca^2+^ concentrations on the growth and cleavage of *B. bacteriovorus FWA*. **(A)** The predation of *B. bacteriovorus FWA* on the host bacteria (*E. coli*) in liquid media under different concentrations of Ca^2+^ is evaluated by changes in OD600, **(B)** *B. bacteriovorus FWA* growth was examined under different concentrations of Ca^2+^, samples were collected at 0, 24, 48, 72, and 96 h from the co-culture of *B. bacteriovorus FWA* with *E. coli,* and *B. bacteriovorus FWA* enumeration was performed via double-agar plate method. All experiments are performed in triplicate, significance analysis uses one-way ANOVA, *p* < 0.05, and the results are the mean value ± SEM.

At 48–72 hpc, in the difference Ca^2+^ superinduce group, OD600 of the co-culture bacterial solution began to decline, particularly with 10 mM, 15 mM, 20 mM, or 25 mM Ca^2+^ superinduce. For the 0 mM superinduce group, OD600 bacterial solution began to decline since 72–96 hpc. This result indicated that appropriate Ca^2+^ superinduce could promote the lysis and growth activity of *B. bacteriovorus FWA*. At 72 hpc, the 25 mM Ca^2+^ superinduce group has the greatest decrease in OD600 bacterial solution, which was 0.54 ± 0.01; and the following were the 15 mM Ca^2+^ superinduce group and 20 mM Ca^2+^ superinduce group, which was 0.53 ± 0.05 (*p* > 0.05) or 0.49 ± 0.04 (*p* > 0.05), respectively. The 0 mM superinduce group has the lowest decrease in OD600 bacterial solution, which was 0.23 ± 0.03 (*p* < 0.05; Figure 7A).

In the 25 mM Ca^2+^ superinduce group, *B. bacteriovorus FWA’s* plaque numbers on double-layer agar medium were fastest. At 48 hpc and 72 h, the number of plaques reached 4.43 ± 0.06 (log, *p* < 0.05) and 5.55 ± 0.04 (log, *p* < 0.05), respectively. For the 30 mM Ca^2+^ superinduce group and 20 mM Ca^2+^ superinduce groups, the number of plaques was 4.42 ± 0.03 (log) and 4.22 ± 0.08 (log) at hpc, respectively. In the 0 mM Ca^2+^ superinduce group, the number of plaques increased at the slowest rate, which was 3.00 ± 0.10 (log) at 72 hpc (Figure 7B).

### 3.9 *Na*^+^ concentration has an influence on *Bdellovibrio bacteriovorus FWA* growth

**Figure 8:**
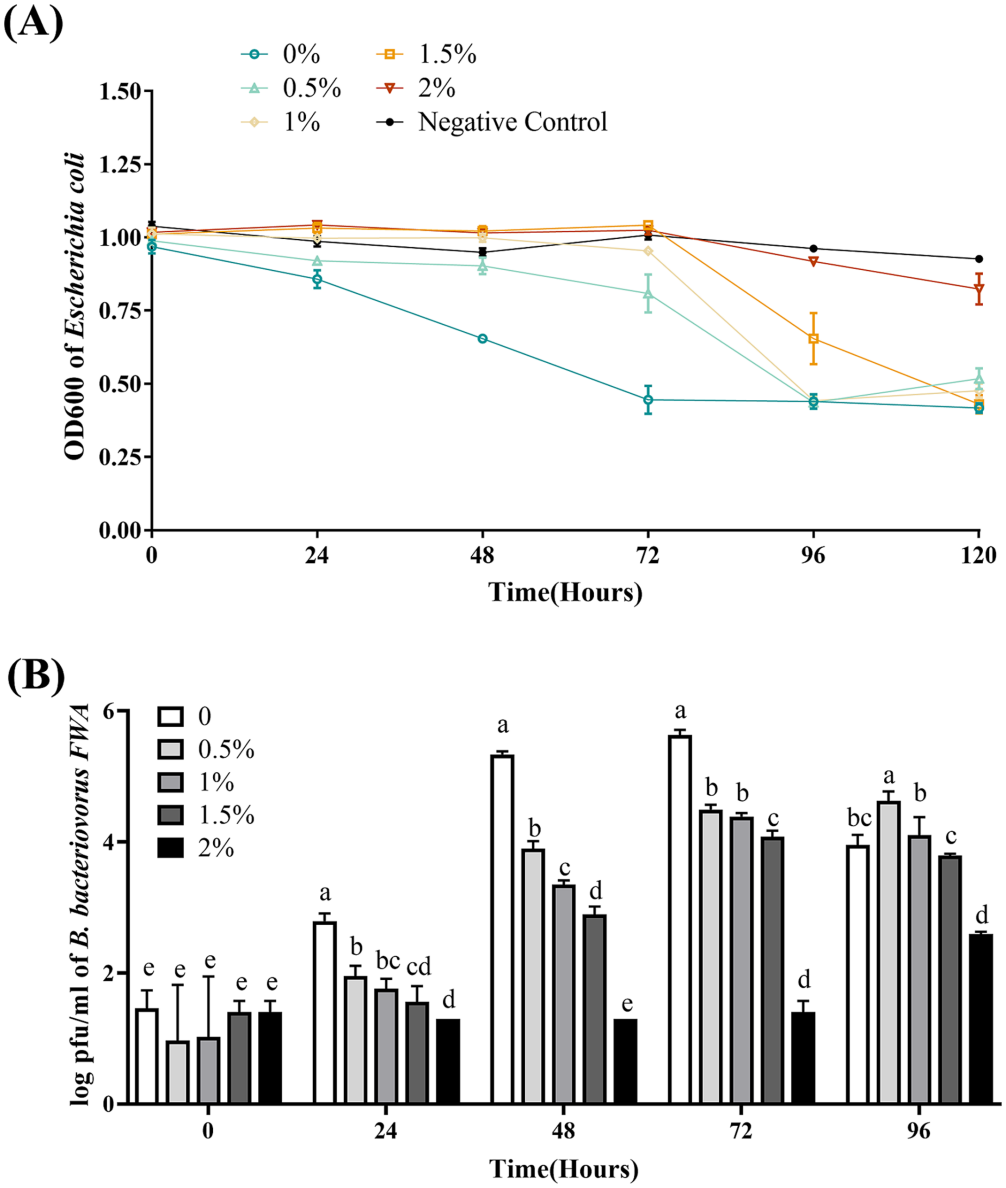
Effect of different Na^+^ concentrations on the growth and cleavage of *B. bacteriovorus FWA*. **(A)** The predation of *B. bacteriovorus FWA* on the host bacteria (*E. coli*) in liquid media under different concentrations of Na^+^ is evaluated by changes in OD600, **(B)** *B. bacteriovorus FWA* growth was examined under different concentrations of Na^+^, samples were collected at 0, 24, 48, 72, and 96 h from the co-culture of *B. bacteriovorus FWA* with *E. coli,* and *B. bacteriovorus FWA* enumeration was performed via double-agar plate method. All experiments are performed in triplicate, significance analysis uses one-way ANOVA, *p* < 0.05, and the results are the mean value ± SEM.

*Bdellovibrio bacteriovorus FWA* showed the best efficiency in *E. coli* lysing without additional Na^+^ addition, where the bacterial solution OD600 began to decrease substantially at 24 hpc–48 hpc, and the bacterial solution OD600 at 48 hpc and 72 hpc was 0.29 ± 0.03 (*p* < 0.01) and 0.50 ± 0.01 (*p* < 0.01), respectively. Under the 0.5 and 1% Na^+^ superinduce, the co-culture bacterial solution of OD600 decreased significantly at 72 hpc–96 hpc, and the OD600 bacterial solution at 96 hpc was 0.55 ± 0.01 and 0.57 ± 0.03, respectively. Under the 2% Na^+^ superinduce, the bacterial solution of OD600 was almost unchanged during the experiment (Figure 8A).

In the absence of supernumerary Na^+^, *B. bacteriovorus FWA* has the best growth status. The number of plaques at 24 hpc and 48 hpc was 2.79 ± 0.07 (log, *p* < 0.01) and 5.33 ± 0.03 (log, *p* < 0.01), respectively. The number of plaques in the 0.5, 1.0, and 1.5% Na^+^ superinduce groups also increased after 48 hpc, while the increase rates were significantly lower than those of group without additional Na^+^ addition. The number of plaques in the 2% Na^+^ superinduce groups also increased during 72 hpc–96 hpc, and the number of plaques at 96 hpc was 2.59 ± 0.02 (log, *p* < 0.01, Figure 8B).

### 3.10 *Bdellovibrio bacteriovorus FWA* reduce the total number of total bacteria and ammonia nitrogen concentration in aquaculture effluent

**Figure 9:**
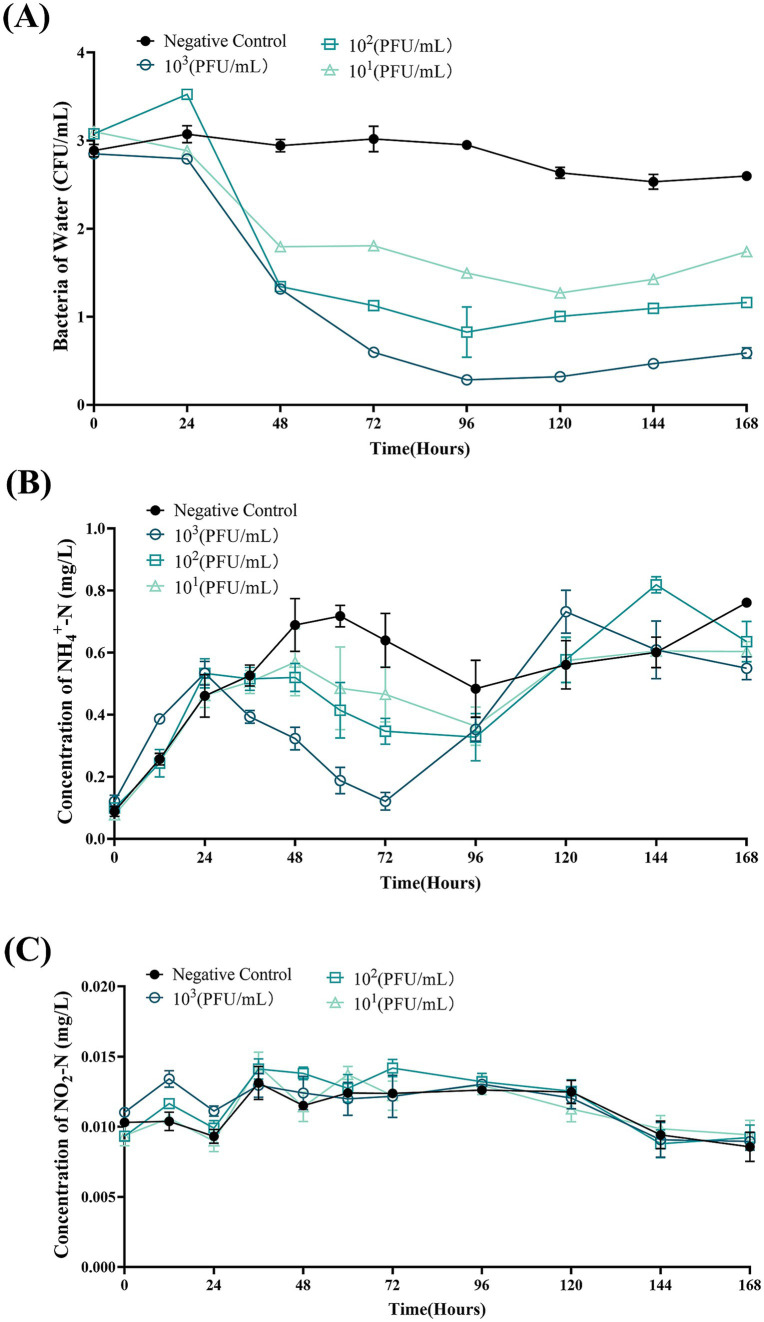
*Bdellovibrio bacteriovorus FWA* lysis of bacteria in aquaculture wastewater and effects on water quality. **(A)** Predation results of different concentrations of *B. bacteriovorus FWA* on bacteria in aquaculture wastewater. **(B)** Effect of *B. bacteriovorus FWA* treatment on NH_4_^+^-N in aquaculture wastewater. **(C)** Effect of *B. bacteriovorus FWA* treatment on NO_2_-N in aquaculture wastewater. All experiments are performed in triplicate, significance analysis uses one-way ANOVA, *p* < 0.05, and the results are the mean value ± SEM.

For the *B. bacteriovorus FWA* with a final concentration of 10^3^ CFU/mL, the total number of bacteria changed most dramatically, and the number of bacteria decreased significantly in the duration of 24–96 hpc. Total bacterial numbers in this group reached the lowest point at 96 hpc, at 0.28 ± 0.01 (log, *p* < 0.05). Compared with the 0 hpc, the total bacteria number reduces by 2.57 ± 0.04 (log). Conversely, the total number of bacteria in the control group rose by 0.12 ± 0.05 (log) at 96 hpc. For the group *B. bacteriovorus FWA* with a final concentration of 10^2^ CFU/mL, the total population of bacteria also reached the lowest point at 96 hpc, at 0.83 ± 0.20 (log, *p* < 0.05). For the group *B. bacteriovorus FWA* with a final concentration of 10 CFU/mL, the total population of bacteria reached the lowest point at 120 hpc, which was 1.27 ± 0.04 (log, *p* < 0.01). The total population of bacteria in the control group did not change significantly during the experiment (Figure 9A).

In the first 24 h of the experiment, the ammonia nitrogen content in each experimental group increased, and then, the ammonia nitrogen content in all groups began to decline. Ammonia nitrogen content in the *B. bacteriovorus FWA* 10^3^ CFU/mL reached the lowest point at 72 hpc, which was 0.12 ± 0.02 mg/L (*p* < 0.01). Except for the negative control group, the other groups also showed an effect on ammonia nitrogen reduction after 24 hpc, and the effect was positively correlated with *B. bacteriovorus FWA* concentration. In the negative control group, the ammonia nitrogen concentrations remained elevated for up to 72 h, peaking at 0.72 ± 0.05 mg/L (Figure 9B). *B. bacteriovorus FWA* treatment did not significantly change the nitrite nitrogen content of each experimental group and the blank control group (Figure 9C).

## 4 Discussion

Aquaculture plays a crucial role in supplying high-quality protein, necessitating its ongoing healthy development. Despite significant progress in the aquatic industry over the years, unresolved issues persist, particularly bacterial diseases. Traditional antibiotic treatments have limitations, and new diseases continue to emerge in aquaculture. Thus, identifying alternatives to antibiotics is essential. In this study, we isolated a strain of *B. bacteriovorus* from freshwater fishpond, designated *B. bacteriovorus FWA*. We analyzed its classification and biochemical properties, as well as various environmental factors, including host bacterial concentration and temperature, that significantly affect its growth. This research aims to provide valuable insights for utilizing BALOs in managing aquatic animal diseases.

Bacterial identification primarily utilizes morphological, physiological, biochemical, and molecular biology techniques. Morphologically, BALOs is classified as a Gram-negative bacterium, observable via electron microscopy, typically exhibiting a curved shape but also presenting as rods, ellipsoids, or comma-like forms (Jafarian et al., 2020). Notably, BALOs possesses an exceptionally long terminal flagellum, measuring 5–40 times the length of the cell body (Thomashow and Rittenberg, 1985). In this study, *B. bacteriovorus FWA* was characterized as accurate with a thicker terminal flagellum, corroborating previous observations. While biochemical identification is crucial for bacterial classification, a standardized approach for identifying BALOs is currently lacking. Consequently, researchers predominantly rely on morphological and molecular biology characteristics to identify BALOs, with only a limited number undertaking biochemical analyses. The biochemical characterization of *B. bacteriovorus FWA* is essential for enhancing the theoretical framework surrounding BALOs.

The 16S rRNA gene, known for its high conservation and specificity among bacteria, serves as a key marker for species identification through sequencing (Yarza et al., 2014). In this study, sequence comparisons revealed that *B. bacteriovorus FWA* exhibited a remarkable 98.89% similarity to *Bdellovibrio* sp. *Ec13*. Furthermore, the *hit* gene, crucial for BALOs’ recognition of host bacteria, has been implicated in phenotypic transitions from host-dependent to non-host-dependent strains due to mutations within its region (Oyedara et al., 2018). Analysis of the *hit* gene in BALOs can facilitate the identification of *B. bacteriovorus* strains. The *hit* gene sequence of *B. bacteriovorus FWA* demonstrated a 95% similarity to *B. bacteriovorus* str. Tiberius and a 94% similarity to *B. bacteriovorus* strain SSB218315, as determined by 16S rRNA sequence analysis. This phenomenon may explain the inability of certain reported strains to amplify the hit gene. In addition, the hit gene appears to be exclusive to *B. bacteriovorus* (Koval et al., 2013; Roschanski et al., 2011), and the amplification failures in some strains may be attributed to variations in predation mechanisms or mutations at this gene locus.

BALOs exhibits a broad host range, effectively lysing nearly all Gram-negative bacteria and certain Gram-positive species. Pineiro et al. (2004) demonstrated that BALOs was particularly potent against various strains, including *Vibrio*, *Escherichia Castellani* and *Chalmers*, *Proteus*, *Salmonella*, *Erwinia*, *Shigella Castellani*, and *Pseudomonas*. While most BALOs species possess a relatively expansive lytic range, variations exist among different species and strains, necessitating the analysis of newly isolated BALOs. The pathogens commonly associated with concentrated aquaculture, such as *E. coli*, *A. hydrophila*, *V. alginolyticus*, *E. tarda*, and *V. parahaemolyticus*, were selected for this study to assess the cleavage characteristics of *B. bacteriovorus FWA*. The findings indicated that *B. bacteriovorus FWA* demonstrated significant lysogenic activity against these five host bacteria, with minimal differences observed in their lysogenicity; however, variations in the timing and size of plaque formation were noted. Notably, larger bacteria such as *E. coli*, *A. hydrophila*, and *E. tarda* exhibited shorter plaque formation times on double-layer agar plates, corroborating the results of Santin et al. (2023), which indicated that larger host cells required longer durations for BALOs division. Consequently, *B. bacteriovorus FWA*, similar to many BALOs strains, is capable of lysing a diverse array of Gram-negative bacteria, surpassing the specificity of phage lysis. Future investigations will further examine the lytic potential of *B. bacteriovorus FWA* against a broader spectrum of Gram-negative and Gram-positive bacteria to enhance understanding of its lytic capabilities.

BALOs, like other organisms, necessitates specific environmental conditions for optimal growth and reproduction. The survival and reproductive requirements of BALOs strains isolated from diverse habitats vary significantly, with extensive research indicating that factors such as host bacterial species, pH, salinity, and temperature are critical for their viability (Williams and Chen, 2020). For host-dependent BALOs, the concentration and type of host bacteria are the primary determinants influencing their growth and reproduction (He, 2016). A 1:10,000 concentration ratio of *B. bacteriovorus FWA* to *E. coli* was identified as particularly conducive for the reproduction of *B. bacteriovorus FWA*. Ottaviani demonstrated that strain HBXCO1 effectively lysed its host and thrived at lower temperatures when maintained at a 1:100 ratio with *V. parahaemolyticus* (Ottaviani et al., 2020). This implies that employing lower quantities of *B. bacteriovorus FWA* could effectively manage pathogenic bacterial proliferation in aquatic environments. In addition, fluctuations in environmental pH influenced bacterial growth and lytic capacity, with *B. bacteriovorus FWA* demonstrating growth and reproduction at pH levels ranging from 5.0 to 10.0 and an optimal pH of 7.0 to 8.0. Previous studies indicated that the preferred pH for terrestrial BALOs is 7.0–7.6, while marine BALOs thrive at 7.2–8.0 (Li et al., 2011). Notably, *B. bacteriovorus FWA* exhibited a broader pH tolerance compared to other BALOs species, suggesting greater ecological versatility. Temperature significantly impacts BALOs survival, with optimal growth for terrestrial BALOs reported between 30 and 37°C and slightly lower for marine BALOs at 20 and 30°C (Williams and Chen, 2020). Excessive temperatures were found to inhibit both growth and lytic capabilities. The optimal temperature range for *B. bacteriovorus FWA* isolated in this study was determined to be 30 to 35°C, aligning with established data for terrestrial BALOs. Salinity also plays a pivotal role in BALOs ecology, genetics, and physiology (Williams and Chen, 2020). The addition of Ca^2+^ was observed to enhance the growth and lytic efficiency of *B. bacteriovorus FWA*. Ca^2+^ facilitates the attachment of BALOs to hosts and promotes its growth within the periplasm. Conversely, a deficiency of Ca^2+^ prolonged the lytic cycle of BALOs. In contrast, the introduction of Na^+^ was found to partially inhibit the growth and lytic activity of *B. bacteriovorus FWA*. While terrestrial BALOs can grow effectively in NaCl-free media, their proliferation is severely restricted at salt concentrations surpassing 0.5%. *B. bacteriovorus FWA*, classified as a molecular biology subgroup of terrestrial BALOs, exhibits Na^+^ sensitivity, further affirming its taxonomic classification.

To assess the practical application value of *B. bacteriovorus FWA*, this study examined its efficacy in lysing bacteria and purifying aquaculture effluent. The results indicated that Gram-negative bacteria in the culture effluent were lysed upon the introduction of *B. bacteriovorus FWA*, with enhanced efficacy observed at higher concentrations, consistent with the findings by Jafarian et al. (2020) and El-Shanshoury et al. (2016). However, complete removal of Gram-negative bacteria was not achieved during the experiment, likely due to the low concentration of host bacteria, which hinders predation by BALOs. The research of Cohen et al. (2021) on the predator–prey interaction between BALOs and host bacteria revealed that BALOs’ abundance fluctuates in relation to prey availability, highlighting the necessity for BALOs to maintain food web stability and species balance while lysing host bacteria. Furthermore, the incorporation of *B. bacteriovorus FWA* into aquaculture effluent demonstrated a regulatory effect on ammonia nitrogen but did not influence nitrite nitrogen. This lack of impact may be attributed to the specificity of *B. bacteriovorus FWA* in targeting certain bacterial populations rather than directly altering nitrogen compounds, or the fed bacterial populations may not significantly affect nitrite nitrogen concentrations. These findings suggest that *B. bacteriovorus FWA* could serve as a viable method for controlling bacterial diseases in aquatic organisms.

## Data Availability

The datasets presented in this study has been deposited in the NCBI GenBank repository (https://www.ncbi.nlm.nih.gov/genbank), accession number OR835679.
